# Integration of existing systematic reviews into new reviews: identification of guidance needs

**DOI:** 10.1186/2046-4053-3-60

**Published:** 2014-06-23

**Authors:** Karen A Robinson, Evelyn P Whitlock, Maya E Oneil, Johanna K Anderson, Lisa Hartling, Donna M Dryden, Mary Butler, Sydne J Newberry, Melissa McPheeters, Nancy D Berkman, Jennifer S Lin, Stephanie Chang

**Affiliations:** 1 Johns Hopkins University, 1830 E. Monument St., Baltimore, MD 21287, USA; 2 Kaiser Permanente Research Affiliates, Portland, OR, USA; 3 Scientific Resource Center for the AHRQ Effective Health Care Program, Portland VA Research Foundation, Portland VA Medical Center, Portland, OR, USA; 4 Evidence-based Practice Center, University of Alberta, Edmonton, AB, Canada; 5 Minnesota Evidence0based Practice Center, Minneapolis, MN, USA; 6 Southern California Evidence-based Practice Center, RAND, Santa Monica, CA, USA; 7 Vanderbilt University Medical Center, Nashville, TN, USA; 8 RTI-University of North Carolina Evidence-based Practice Center, Research Triangle Park, NC, USA; 9 Center for Outcomes and Evidence, Agency for Healthcare Research and Quality, Rockville, MD, USA

**Keywords:** systematic review methods, evidence-based practice centers, using existing reviews

## Abstract

**Background:**

An exponential increase in the number of systematic reviews published, and constrained resources for new reviews, means that there is an urgent need for guidance on explicitly and transparently integrating existing reviews into new systematic reviews. The objectives of this paper are: 1) to identify areas where existing guidance may be adopted or adapted, and 2) to suggest areas for future guidance development.

**Methods:**

We searched documents and websites from healthcare focused systematic review organizations to identify and, where available, to summarize relevant guidance on the use of existing systematic reviews. We conducted informational interviews with members of Evidence-based Practice Centers (EPCs) to gather experiences in integrating existing systematic reviews, including common issues and challenges, as well as potential solutions.

**Results:**

There was consensus among systematic review organizations and the EPCs about some aspects of incorporating existing systematic reviews into new reviews. Current guidance may be used in assessing the relevance of prior reviews and in scanning references of prior reviews to identify studies for a new review. However, areas of challenge remain. Areas in need of guidance include how to synthesize, grade the strength of, and present bodies of evidence composed of primary studies and existing systematic reviews. For instance, empiric evidence is needed regarding how to quality check data abstraction and when and how to use study-level risk of bias assessments from prior reviews.

**Conclusions:**

There remain areas of uncertainty for how to integrate existing systematic reviews into new reviews. Methods research and consensus processes among systematic review organizations are needed to develop guidance to address these challenges.

## Background

Evidence-informed decisions in health care rely on the explicit consideration of available evidence through the conduct of systematic reviews. In the context of exponential increase in the number of systematic reviews published, constrained resources for new reviews, and the increase in the need to update reviews, there is an urgent need for detailed methods to explicitly and transparently integrate existing reviews into systematic reviews.

Since 1997, the US Agency for Healthcare Research and Quality (AHRQ) has supported the development of systematic reviews about health care through its Evidence-based Practice Center (EPC) program. These reviews generally address multiple questions and are conducted following standard methods. A number of years ago, researchers from several EPCs recognized that reviewers were increasingly faced with decisions about whether and how to incorporate existing systematic reviews into new systematic reviews. This group collaborated to develop guidance for the use of existing systematic reviews in new reviews [1]. As part of the work in developing the EPC Methods Guide, a workgroup was subsequently formed by the AHRQ on the same topic, and guidance that generally reflected the prior work was issued [2].

The guidance we provided in the EPC Methods Guide noted the need for additional work on specific methods for using existing systematic reviews in reviews. Our intervening experience in implementing this guidance has also identified additional challenges, particularly in grading the strength of evidence and presenting the body of evidence when existing systematic reviews form part of the overall evidence base in a new systematic review.

The lack of guidance has led to variable perspectives about and usage of existing systematic reviews across the EPC program. In particular, uncertainty and lack of guidance has led some EPC researchers to avoid integrating existing systematic reviews within the strength of evidence judgments and summaries in new reviews. Omitting existing reviews from consideration when assessing the strength of the evidence is unsatisfying for the EPCs and for the audiences of our reports, as recently noted in recent feedback by the Eisenberg Center [3]. Some EPCs have chosen to integrate existing systematic reviews into the strength of evidence ratings, but without guidance on how to implement such integration, the process has been inconsistent.

The objectives of our workgroup of EPC members, and of this white paper, are: 1) to identify areas where existing guidance may be adopted or adapted, and 2) to suggest approaches and focused areas for future work and additional guidance needs.

## Methods

**Figure 1:**
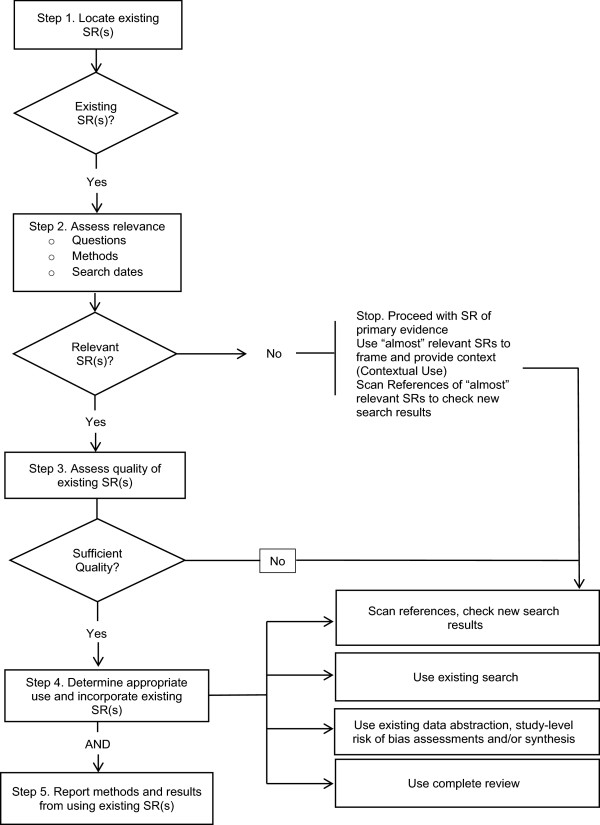
Methodological steps in using existing systematic reviews (SRs).

Key methodological steps in deciding whether and how to use existing systematic reviews in new systematic reviews of health care questions are illustrated in Figure 1 and in the accompanying Table 1 (adapted from Whitlock *et al*. [1]). These steps include five, non-mutually exclusive options for how existing systematic reviews might be used. In this paper, these methodological areas were used to classify existing guidance from other health-focused systematic review organizations and to focus the discussion with directors and staff at EPCs. Table 1 provides definitions for the key steps in Figure 1.

**Table 1:** **Definitions of terms used in Figure**1

**Locate existing SR(s)**
A defined and reproducible approach to efficiently identify existing systematic reviews for possible use in conducting a newly proposed systematic review, including updates.
**Assess relevance**
Methods by which existing systematic reviews identified in Step 1 can be evaluated as to whether they are similar enough to the newly proposed review to obviate the need for conducting one or several steps in undertaking the newly proposed review. Relevance evaluation considers how well the existing reviews’ research questions and inclusion/exclusion criteria for population, interventions, comparators, outcomes, settings, and study designs match those of the new systematic review, and how recently existing reviews’ literature searches were concluded. Use ‘almost’ relevant SR(s) when selecting, developing, and/or refining questions and providing context for a newly proposed systematic review (that is, Contextual Use) and scan references to check new search results.
**Assess quality of existing SR(s)**
Methods by which relevant existing systematic reviews can be evaluated for quality of methodological approach, using AMSTAR or other commonly used tools, with a focus on potentially incorporating only reviews meeting certain quality criteria into the proposed review [4,5].
**Determine appropriate use and incorporate existing SR(s)**
Methods by which to determine appropriate uses for relevant, high-quality existing systematic reviews in the proposed review. Incorporate existing SR(s) or use information from existing systematic reviews to supplement or supplant one or more activities that would be conducted from scratch for the newly proposed review. Use of existing systematic reviews may include: 1) using the existing systematic review(s)’ listing of included studies as a quality check for the literature search and screening strategy conducted for the new review (Scan References); 2) using the existing systematic review(s) to completely or partially provide the body of included studies for one or more key questions in the new review (Use Existing Search); 3) using the data abstraction, risk of bias assessments, and/or analyses from existing systematic reviews for one or more key questions in the new review (Use Data Abstraction/Syntheses); or 4) using the existing systematic review(s), including conclusions, to fully or partially answer one or more key questions in the new review (Use Complete Review).
**Report methods and results from using existing SR(s)**
Standards for reporting the rationale for incorporating existing systematic reviews and the methods by which specific existing systematic reviews were located, assessed, and incorporated into the current systematic review. Standards for reporting results in the current systematic review that rely on evidence and/or analysis from existing systematic reviews and caveats or limitations associated with that approach; guidance about discussing how findings of the current systematic review compare and contrast with those of existing relevant systematic reviews.

AMSTAR, A Measurement Tool to Assess Systematic Reviews; SR, systematic review. Adapted from Whitlock *et al*. [1].

### Guidance summary

Documents and websites from healthcare focused systematic review organizations were manually searched for available guidance on the use of existing systematic reviews in new systematic reviews. Where available, relevant guidance was extracted and summarized for each of the methodological steps illustrated in Figure 1.

### Evidence-based practice center discussions

Individual workgroup members held discussions with volunteer EPC members including EPC directors, associate directors, and project managers. An interview guide, including 11 open-ended questions, facilitated the informal discussions. The goals of the discussions were to gather insights from the experiences of EPCs in integrating existing systematic reviews into new reviews and to identify common issues and challenges, as well as potential solutions. Individual workgroup members conducted discussions with 15 EPC members representing 10 of 11 EPCs. A list of participating EPC members can be found in Appendix A. Each call was, with permission of those on the call, recorded and then transcribed. Major themes from the interviews were identified by workgroup members through review of the transcripts. These themes were then compared to the existing guidance to identify areas of overlap and need.

### Assessment of areas of need

Workgroup members assessed the sufficiency of currently available guidance based on the needs expressed by EPCs. Through individual and group deliberation, the workgroup determined which methodological steps on the use of existing systematic reviews need additional guidance. For each area where additional guidance is needed, the workgroup generated suggestions for how to develop this guidance.

## Results

### Guidance summary

Overall, about half of the systematic review organizations provided some direction on the use of existing systematic reviews in new reviews. Table 2 shows the list of organizations for which websites were manually searched. A check mark under the area of guidance denotes a comment on the use of existing systematic reviews, though not necessarily detailed guidance. Listed organizations with no check marks are organizations for which guidance on the use of existing systematic reviews was not found.

**Table 2:** Available guidance/comment on the use of existing systematic reviews

	**Guidance areas**								
	**Locating and defining appropriate use**	**Assessing relevance**	**Assessing review quality**	**Determining use: Scanning references**	**Determining use: Search strategy**	**Determining use: Risk of bias assessment**	**Determining use: Data abstraction**	**Determining use: Synthesis**	**Methods/Results reporting**
**AHRQ Evidence-based Practice Center Program (EPC program)**	✓	✓	✓	✓	✓	✓	✓	✓	✓
*Methods Guide for Effectiveness and Comparative Effectiveness Reviews*[2]									
*Reporting the Findings of Updated Systematic Reviews of Comparative Effectiveness*[6]									✓
**Canadian Agency for Drugs and Technology in Health (CADTH)**									
*Guidelines for Authors of CADTH Health Technology Assessment Reports*[7]									
**Cochrane Collaboration**	✓	✓	✓	✓	✓	✓	✓	✓	✓
*Cochrane Handbook for Systematic Reviews of Interventions*[8]									
**Danish Centre for Health Technology Assessment (DACEHTA)**	✓	✓				✓			
*Health Technology Assessment Handbook*[9]									
**European Collaboration for Health Technology Assessment (ECHTA)**	✓	✓				✓			
*Best Practice in Undertaking and Reporting Health Technology Assessments*[10]									
**Health Technology Assessment International**									
**Institute of Medicine**									
*Finding What Works in Health Care: Standards for Systematic Reviews*[11]									
**Institute for Quality and Efficiency in Health Care (IQWiG)**	✓		✓						✓
*General Methods*[12]									
**National Institute for Health and Care Excellence (NICE)**	✓		✓				✓		✓
*The Guidelines Manual, Draft for Consultation*[13]									
**York Centre for Reviews and Dissemination: Systematic Reviews (CRD)**	✓		✓	✓					
*CRD’s Guidance for Undertaking Reviews in Healthcare*[14]									

### Evidence-based practice center discussions

Overall themes were identified from the discussions with EPCs:

• EPCs most commonly used existing reviews as a source of relevant literature and as context for the introduction or discussion section of reviews.

• Existing reviews were most useful when key questions and/or PICOTS-SD (population, intervention, comparator, outcome, time frame, setting, and study design) matched or when they addressed a specific subquestion of the new review.

• Using existing reviews was often more resource intensive than completing a review from scratch.

• EPCs expressed that they often did not trust aspects of reviews conducted by others.

• When relevant and rigorous, incorporating prior reviews into the review being undertaken by the EPC was clearly valuable in at least two instances: 1) allowing larger scope of the review being undertaken without additional resources, or 2) providing summarized evidence when a new in depth review of primary literature would not be feasible (for example, existing reviews provide individual patient data analysis or include hundreds of trials, supplemented by author-provided data).

EPCs have used existing reviews in various ways, most commonly as a source of relevant literature, allowing them to reduce the extent of searching to locate primary literature or to check completeness of primary literature search strategies. Additionally, prior reviews are often used to provide context for the introduction or discussion sections of a review. At a minimum, most EPC members feel that it is necessary to acknowledge other systematic reviews and to put the findings of the current review into the context of other systematic reviews, particularly in the case of disagreements or controversy.

EPC members noted that methods determining when and how to use an existing review are highly dependent on the topic and scope of the new report. There are certain instances when it may be most feasible to use an existing review as evidence in a new review. For example, existing reviews are more likely to be used as evidence for a new review when the key questions match and when the review questions are concerned primarily with one or a few clearly and specifically defined and measured outcomes; existing systematic reviews are fruitful to incorporate to answer contextual questions that do not demand a full systematic review, but are important for decision makers when considering the evidence base. Conversely, EPC members indicated there are other instances when it would not make sense to use an existing review, such as when there are few studies on a given topic or when there are multiple conflicting systematic reviews. When incorporating existing reviews into a new or updated review, EPC members most often described qualitative or narrative incorporation of the existing reviews, noting that quantitative combination of findings (without going back to all primary studies) is more difficult and potentially introduces error, and thus, is less commonly done.

EPC members varied in their thoughts and experiences about the efficiency of incorporating existing reviews into new reviews. Most thought that, although this process theoretically should result in efficiency gains, challenges that arise when trying to use reviews often negate any increased efficiency. These challenges arise from the need to understand and qualify the methods for existing systematic reviews as intensively as primary studies. Thus, EPC members often described experiences where using a prior systematic review resulted in as or more intensive resource requirements as completing a new review of primary literature. A particular concern were instances in which stakeholders who nominated the review requested a new review after seeing the results of incorporation of existing reviews. It is hard to estimate how much work will be required to clarify the relevance or quality of existing systematic reviews, since effort depends on the volume of existing systematic reviews, as well as issues specific to the match between existing reviews and the review being undertaken; these include whether the key questions are an exact match or how the existing systematic review authors approached important methods, such as strength of evidence grading or risk of bias assessment. In a number of cases, EPC members described eventually having to conduct much of the process from scratch in spite of completing the additional step of in-depth examination of existing reviews. EPC members voiced concern about using this process based on the potential (although often unrealized) benefit of efficiency, while running an unclear risk of error or reduced quality from relying on unverifiable work from others. Additionally, EPC members described discomfort with the lack of guidance in this area, noting inconsistency across EPCs in how this process is approached and hesitancy to engage in such a process without explicit direction in the EPC Methods Guide.

Although EPC members felt that it was often difficult to gain efficiency incorporating existing reviews in new reviews, they acknowledged other potential benefits to this process. Some described that including existing reviews sometimes enables them to cover a wider range of questions and elements of questions (as denoted by PICOTS) when existing systematic reviews address important aspects of new review key questions. EPC members suggested that this breadth is often desirable, but may not be possible if independent review of the primary literature is required due to time and resource limitations. Similarly, important evidence to answer some key questions may include individual patient data meta-analyses or data from a large number of trials, representing work from systematic reviewers that would be very difficult or impossible to reproduce.

Overall, EPC members noted that more guidance is needed for using existing reviews in new systematic reviews. Most felt that detailed, specific, step-by-step guidance may not be feasible, but that some further articulation of important areas to consider, with clear worked examples, would be helpful. Commonly highlighted areas in need of additional guidance include:

1. Providing principles or criteria for when a new review adds value to a field with many existing reviews.

2. Providing templates or advisory considerations for construction of evidence tables for reviews combining primary and secondary (systematic review-level) evidence.

3. Reporting guidelines for clearly communicating the methods for locating, selecting, and deciding how best to utilize existing systematic reviews.

4. Methods that limit the potential for bias in selecting reviews to incorporate from among multiple existing reviews.

5. Guidance on methods that limit the potential for bias in incorporating selected portions of a review.

6. Qualitative and quantitative methods for summarizing bodies of evidence that include a systematic review as the only or as one source of evidence.

7. More robust means for quality rating of existing systematic reviews (beyond AMSTAR [4]).

8. Specific methods to grade strength of evidence for bodies of evidence that include a systematic review as the only or as one source of evidence.Guidance addressing the first item listed is more of a scoping question prior to the initiation of a new review (for example, within the Topic Refinement stage of the EPC program’s current processes). In this paper, we assume that a new review has been started and is the review authors are considering incorporating existing reviews. The remaining challenges in using existing reviews are discussed below and fall within each of the methodological areas presented in Figure 1. A summary of the existing guidance for each area is presented along with an assessment of future guidance needs.

## Discussion

### Methodological areas: assessment of areas of need

#### Locating existing systematic reviews

##### Available guidance

Several organizations present guidance on locating existing systematic reviews, including the EPC program and the Cochrane Collaboration (see Table 3). These groups recommend using specific databases and search filters to aid in locating existing systematic reviews. Commonly recommended databases include: Database of Abstracts of Reviews of Effects (DARE), Cochrane Database of Systematic Reviews (CDSR), Health Technology Assessment Database, MEDLINE and Embase. Some organizations promote limiting searches for existing systematic reviews to selected sources (for example, CDSR), with the idea that these systematic reviews would be expected to meet sufficient quality standards. We identified no criteria for selecting systematic reviews.

**Table 3:** Guidance summary

	**AHRQ Evidence-based Practice Center Program (EPC program)**		**Cochrane collaboration**	**Danish Centre for Health Technology Assessment (DACEHTA)**
Locating	Two strategies are recommended for identifying existing systematic reviews for a CER. The first strategy is to perform a targeted search of a higher yield database, which includes output from the Evidence-based Practice Center program, MEDLINE’s Top 120 Index Medicus Journals, Health Technology Assessments, Cochrane Database of Systematic Reviews and Database of Abstracts and Reviews of Effects. The second strategy is to identify systematic reviews during a broad *de novo* literature search.		Systematic reviews can be located through CDSR, DARE and HTA database. MEDLINE and EMBASE can also be used to search for systematic reviews. In MEDLINE, most review articles can be found under the publication Term ‘Meta-analysis’ and in EMBASE, the thesaurus term ‘Systematic Review’ can be used. Specific search strategies can be used to identify systematic reviews in MEDLINE and EMBASE. Additionally, systematic reviews can be identified through search services such as Turning Research into Practice (TRIP).	Secondary studies (for example, systematic reviews, HTA reports, and clinical guidelines) should be located to determine if key questions have already been answered. Secondary studies can be identified through several databases (for example, The HTA Database, Cochrane Database of Systematic Reviews, Database of Abstracts of Reviews of Effects, Guidelines International Network, National Guidelines Clearinghouse, Health Evidence Network, National Electronic Library for Health: Guidelines Finder, and Turning Research Into Practice).
			In an Overview, primarily only Cochrane Intervention reviews should be included, but other reviews may be included occasionally	
Assessing Relevance	An existing systematic review should be used with the intent to answer parts or all of specific key questions. PICOTS-SD must be considered for relevance of existing systematic reviews. Reviews that are partially relevant may be useful for background or checking references. An initial screening for relevance should be performed, considering the timeliness of the review’s literature search. It is recommended to bridge any search date that ended more than one year from the time the systematic review is identified. If a review is outdated but still desired to be used, an update of the search should be done.		In an Overview, included reviews should be assessed using specific criteria. Considerations include whether a review is up-to-date and if there are specific limitations for the objectives of the Overview.	All evidence should be assessed for relevance to the topic. Identified articles should be compared to the focused question to determine if the article may answer the focus question. The literature can be divided into two groups (secondary studies and primary studies). If a large amount of evidence has been identified, it can be subdivided into groups based on presumed quality. The hierarchy of evidence is: 1) meta-analyses and systematic reviews (among others Cochrane reviews); 2) randomized controlled trials (RCTs); 3) non-randomized controlled trials; 4) cohort studies; 5) case-control studies; 6) descriptive studies, limited series; and 7) position papers, non-systematic reviews, leading articles, expert opinions.
	In the second stage of screening, the review’s PICOTS-SD elements should be compared to those in the new review protocol for relevance. If these elements are poorly reported, the review should not consider including the existing review.			
Assessing Review Quality	Only reviews of high quality should be included in a CER. Two independent reviewers should assess for quality and methods for resolving discrepancies should be reported. A quality rating instrument should be used to addresses all aspects of the review that will be incorporated into the CER. Both the methods used to minimize bias and the reporting should be assessed. QUOROM (PRISMA) is a checklist that can be used to assess the reporting of systematic reviews. As a common starting point, the AMSTAR tool should be used to assess the quality of reviews. Reproducibility and application of inclusion and exclusion criteria should be confirmed. As some limitation to AMSTAR exists, it is recommended to describe implications of potential methodological flaws instead of relying on numerical scores.		Generally, selection criteria for a Cochrane Overview limits included reviews to Cochrane reviews. Non-Cochrane systematic reviews may be included if there are good quality reviews for which a Cochrane review is not available.	No guidance
Determining use: Scanning References	The list of included articles from an existing review can be used in a CER if methods for identifying articles are of adequate quality.		Existing systematic reviews can be used as sources of relevant studies. References lists of systematic reviews can be searched to identify relevant articles. This should be done as an adjunct to other search methods as bias may be present in what studies were included in existing reviews.	No guidance
Determining use: Use Search	Part or all of the search strategy may be used from an existing review if it is consistent with EPC program methods for finding evidence. A search strategy from an existing review can be used, followed by *de novo* analysis and synthesis of data.		Existing reviews may be a useful source information about search strategies	No guidance
Determining use: Risk of Bias Assessment	In order to use the risk of bias assessment from a systematic review, the methods used must be consistent with the EPC program methods guide. These methods include selection of design specific criteria for risk of bias assessment and use of appropriate tools.		In an Overview, an assessment of the quality of evidence should be done. If no assessment was done in an included systematic review, authors should perform the assessment. If a quality assessment was done in an included systematic review, authors should assess the judgments and ensure consistency between included reviews.	A quality assessment tool should be used to uniformly assess the quality of identified articles. Check list tools developed by different national centers (for example, SIGN, NICE, GRADE and Centre for Evidence-based Medicine, Oxford) can be used to assess quality.
Determining use: Data Abstraction	The data extraction tables may be used from an existing review, if they are deemed to be of adequate quality. However, if results of individual trials are not reported, the use of summary findings from an existing review may compromise transparency for a CER and this is not recommended.		In an update, data from new studies should be abstracted and included, if applicable.	No guidance
			In an Overview, if necessary, authors may seek additional data or information from the authors of primary studies from included systematic reviews.	
Use Synthesis	If an existing review is very similar to a CER in research questions and is high quality, the entirety or portions of the existing review may be incorporated. Summarized evidence for specific populations or interventions may be included in a CER. If summarized evidence is to be included, the existing review must have methods consistent with EPC program methods for finding evidence, assessing quality, grading the strength of evidence and other principles including conflicts of interest. Summarized evidence can also be incorporated with a *de novo* sensitivity analysis.		In an update, data collected from new studies should be included and a new meta-analysis should be done.	No guidance
	If multiple high quality reviews are found, a single review can be chosen, which is most relevant and least biased, or a meta-review can be performed.		In an Overview, authors should reply on previous analyses when possible. If there are differences between reviews (for example, different populations or subgroups are analyzed), data may need to be reanalyzed.	
	If more than one high quality review is found with discordant findings, it may be an indication to start a *de novo* review on that key question.			
Report Methods/Results	It is recommended to provide a summary table to show where existing review(s) were used to replace *de novo* processes. Summary tables of existing systematic reviews should be included to compare the reviews and should address any overlap (or lack thereof) in the primary research included in reviews.		In an update, revision to text of the existing review will depend on the influence of the new data and results. If there is no change in the results, little revision to the text is required. However, some updates may require a change to the conclusion of a review which will require much modification of the text. It should be noted in the Abstract and Background that this is an update. A ‘What’s new’ table should be completed and changes should be made to ensure no dates or other information is out of date.	No guidance
	The discussion section should include a justification for using an existing systematic review and address any limitations. It is also important to compare findings from the CER with the findings from existing reviews.			
	In an update, it is important to show explicitly what has changed from the previous report. The desired depth of information varies between users of reports. Review updates may be effectively presented in an executive summary with tables and figures, identifying and modifications followed by a full report for users who require further depth of information.			
	**European Collaboration Health Technology Assessment (ECHTA)**	**Institute for Quality and Efficiency in Health Care (IQWiG)**	**National Institute for Health and Clinical Excellence (NICE)**	**York Centre for Reviews and Dissemination: Systematic Reviews (CRD)**
Locating	In order to determine if key questions have already been answered, a search for previous HTA reports should be conducted. The search for previous reports should be systematic and well documented.	Different databases should be considered in locating systematic reviews that are used for primary literature. Databases that exclusively or mostly hold systematic reviews should be searched as well as biomedical databases (for example, MEDLINE and EMBASE), which also hold systematic reviews.	Core and subject databases, including MEDLINE, EMBASE, Cochrane Database of Systematic Reviews, Database of Abstracts of Reviews of Effects, Cochrane Central Register of Controlled Trials, and Health Technology Assessment (HTA) database, should be searched for every review. For questions on effectiveness, a search should be done for systematic reviews, followed by randomized controlled trials, then cohort or case–control studies. Search filters are available to assist in identifying studies, including a search filter for systematic reviews.	To check if key questions have been answered by existing or ongoing reviews, a search for systematic reviews should be conducted. The Database of Abstracts of Reviews of Effects (DARE), and the Cochrane Database of Systematic Reviews (CDSR) should be searched. Additionally, NICE and NIHR HTA program Websites can be searched along with the Campbell Library of Systematic Reviews and the Evidence for Policy and Practice Information Centre’s Database of Systematic and Non Systematic Reviews of Public Health Information (DoPHER). Guideline groups including NGC and SIGN can be searched for guidelines based on systematic review evidence. MEDLINE and other databases can be searched for previous reviews.
		Evidence scanning should be done continuously to identify systematic reviews that concern published or developing information products. Two people should regularly screen (CDSR, DARE, INAHTA, MORE and PubMed). Identified reviews that concern a product of the Institute can influence the updating process, including triggering an update or modifying the updating plan.		
Assessing Relevance	All identified evidence should be assessed with pre-defined inclusion and exclusion criteria. Selection criteria should be developed from background information, research questions and the availability of evidence and should be defined prospectively to avoid bias in selection of evidence. Inclusion and exclusion criteria should cover patient characteristics, condition characteristics, technology aspects, methodological issues, outcomes measured, publication type.	No guidance	No guidance	No guidance
Assessing Review Quality	No guidance	For systematic reviews to be used in a benefit assessment, they must be assessed for sufficient quality. They must ‘show only a minimum risk of bias; present the evidence base in a complete, transparent and reproducible manner; and thus allow clear conclusions to be drawn’. The searches conducted in the systematic reviews must not contradict the methodology of the Institute. Quality assessment should be done with Oxman and Guyatt’s quality index for systematic reviews or AMSTAR. Sponsors and authors’ conflicts of interests should be documented and discussed for systematic reviews.	Guidelines may contain reviews of evidence that are applicable to questions formulated by the guideline development group. These may be used as evidence if: ‘they are assessed using the appropriate methodology checklist from this manual and are judged to be of high quality, they are accompanied by an evidence statement and evidence table(s), the evidence is updated according to the methodology for the exceptional update of NICE clinical guidelines’.	Identified reviews should be assessed for quality. Quality reviews should have a well-defined question, comprehensive search, clear and appropriate selection or studies, unbiased processes for assessing study quality and extracting and synthesizing data. Checklists can be used to help in assessing quality of systematic reviews (for example, Oxman and Guyatt, Guidelines for reading literature reviews).
		If more than one systematic review of adequate quality is found to address a particular subject, additional quality assessment should be done. Items to compare include: content of the review, search strategy and date, sensitivity analysis, how bias is assessed and dealt with, and updating provisions.		
Determining use: Scanning References	No guidance	No guidance	No guidance	Other sources of literature include references lists of systematic reviews. References lists of existing reviews can be scanned to identify additional studies.
Determining use: Search	No guidance	No guidance	No guidance	No guidance
Determining use: Risk of Bias Assessment	All evidence should be critically assessed for quality. Checklists can be used for appraisal of medical literature. All sources of information should be appraised for validity. No guidelines exist for assessing quality of sources of information other than medical literature, and this is a gap that future guidance needs to address.	No guidance	No guidance	No guidance
Determining use: Data Abstraction	No guidance	No guidance	If using reviews of evidence published in other guidelines, the guideline development group should create new evidence summaries or statements. The original evidence tables should be referenced with a direct link to the source if possible or a reference to the published document. Verbatim quotes of recommendations from other guidelines should not be used, unless the recommendations come from NHS policy or legislation.	No guidance
Determining use: Synthesis	No guidance	No guidance	No guidance	No guidance
Report Methods/ Results	No guidance	Results of systematic reviews should be summarized in tables if possible. If discordant results on the same outcome are found, possible explanations should be given. If it appears that a new benefit assessment based on primary studies would produce different results, a new assessment should be done.	Original evidence tables from published guidelines should be referenced with a direct link to the source if possible or a reference to the published document. Verbatim quotes of recommendations from other guidelines should not be used, unless the recommendations come from NHS policy or legislation.	No guidance

CDSR, Cochrane Database of Systematic Reviews; CER, comparative effectiveness review; DARE, Database of Abstracts of Reviews of Effects; DoPHER, Database of Systematic and Non Systematic Reviews of Public Health Information; GRADE, Grading of Recommendations Assessment, Development and Evaluation; INAHTA, International Network of Agencies for Health Technology Assessment; MORE, McMaster Online Rating of Evidence; NIHR, National Institute for Health Research; NGC, National Guide Clearinghouse; NHS, National Health Service; NICE, National Institute for Health and Care Excellence; PICOTS-SD, population, intervention, comparator, outcome, time frame, setting, and study design; PRISMA, Preferred Reporting Items for Systematic Reviews and Meta-Analyses; QUOROM, Quality of Reporting of Meta-analyses; SIGN, Scottish Intercollegiate Guidelines Network; TRIP, Turning Research into Practice.

##### Evidence-based practice center discussions

EPC members expressed concerns about locating reviews based on very limited sources, for example, only searching for EPC or Cochrane reviews. Some EPC members were uncomfortable with any type of selective search instead of doing a broader, if not comprehensive, search for existing systematic reviews. However, other EPC members felt it could be appropriate to selectively use one or two earlier reviews without having to review all of the available prior reviews, pointing out that the scientific rationale and purpose in conducting a systematic search for existing systematic reviews is different than when searching for primary studies. Currently, there is a lack of consensus and limited guidance concerning how to adequately locate and transparently select and use only a subset of reviews.

##### Assessment

Current EPC guidance on locating existing systematic reviews states that EPCs should conduct a targeted search of a higher yield database, which includes output from the EPC program, MEDLINE’s Top 120 Index Medicus Journals, Health Technology Assessments, CDSR and DARE. EPC program guidance suggests that identifying existing systematic reviews may be done separately or be completed as part of the broad search for primary literature to answer key questions. EPC members shared concern about the extensive effort that may be required to search for and locate all reviews and assess their quality. Instead, given the purpose of locating existing systematic reviews, the ideal search might locate only highly relevant, well-done, very recent, existing systematic reviews that are more likely to allow the current reviewer to leverage the prior work. Some members proposed that future guidance further limit the search for earlier reviews to sources that may have greater likelihood of identifying higher quality and better reported reviews, such as the EPC and Cochrane databases. This would also, theoretically, negate the need to determine if a report is a systematic review.

#### Assessing relevance

##### Available guidance

Several organizations present guidance on assessing the relevance of existing systematic reviews. European Collaboration for Health Technology Assessment (ECHTA) and Danish Centre for Health Technology Assessment (DACEHTA) provide general guidance for assessing the relevance of all literature, but no guidance specific to existing reviews. Cochrane provides guidance for assessing the relevance of existing systematic reviews for use in an overview of reviews and specifically recommends that reviewers consider if a review is up-to-date (up-to-date is not defined in this context, but for Cochrane reviews is considered as a review with a search conducted within last 2 years). The EPC program provides EPCs with some guidance for assessing whether an existing review can answer key questions or sub-questions of a review. The EPC program recommends consideration of how recently the review’s primary literature search ended and how well the PICOTS-SD elements are reported and match with the current review, noting that partially relevant reviews may be useful for background or checking references. As described in Table 3, specific steps recommended in EPC guidance include conducting an initial screening for relevance while considering the timeliness of the review’s literature search. In the second stage of screening, the review’s PICOTS-SD elements should be compared to those in the new review protocol for relevance. If these elements are poorly reported, the new reviewers should not consider incorporating the existing review. If a review is outdated, but otherwise on point, an update of the search could be done.

##### Evidence-based practice center discussions

EPC members raised concerns about deciding which existing reviews, if any, to use in a new review, especially in situations when many reviews of varying quality or scope are available. It can often be difficult to find reviews that match all elements of question. Still, existing reviews may be sufficiently similar to consider using, although criteria for all situations are not easy to define. Thus, determining which reviews are ‘close enough’ is inherently subjective and can be time consuming.

##### Assessment

Guidance exists for assessing the relevance of existing reviews for conducting overviews of reviews and for integrating existing reviews into a new review. Even so, EPC members expressed concerns about selecting which reviews to use when many are available. They reported rarely finding reviews that match directly all elements of questions. A clearer understanding of the factors (such as clinical area, review purpose, volume of literature, type of review) that have been correlated with successful (and unsuccessful) attempts to incorporate prior systematic reviews into new reviews would be helpful.

#### Assessing review quality

##### Available guidance

Guidance is available on assessing the overall quality of systematic reviews (see Table 3). ECHTA and DACEHTA provide general guidance for assessing the quality of all literature, but do not specifically address assessing the quality of systematic reviews. In contrast, Institute for Quality and Efficiency in Health Care (IQWiG) and York Centre for Reviews and Dissemination (CRD) present guidance on assessing the quality of systematic reviews and recommend the use of a quality rating tool or checklist such as Oxman and Guyatt’s quality index or the AMSTAR Instrument [4,5]. NICE suggests using existing reviews as evidence in developing a new guideline if they are up-to-date, have been assessed as high quality using the NICE methodology checklist for systematic reviews [15], and include the accompanying evidence statements and evidence tables. EPC guidance recommends using AMSTAR as a starting point for quality assessment and supplementing as deemed appropriate for specific reviews. The Cochrane Collaboration is currently developing a new tool (ROBIS) to assess risk of bias in systematic reviews. In addition, the IOM report *Finding What Works in Health Care: Standards for Systematic Reviews* may provide the basis for the development of instruments to assess quality of systematic reviews [11].

##### Evidence-based practice center discussions

EPC members raised concerns about using AMSTAR to rate the quality of reviews, specifically, as to how accurate AMSTAR is in differentiating between poorer quality and better quality reviews. This is important since EPCs generally agreed that evidence from existing reviews should only be used if the review can be relied upon to substitute for the methods for the conduct of reviews espoused by the EPC program. A suggestion was raised for a minimum standard of quality to be set for earlier reviews that are included as evidence in new reviews.

##### Assessment

Guidance is available for quality rating of reviews with available tools and checklists (for example, Oxman and Guyatt, AMSTAR, and National Institute for Health and Care Excellence (NICE)) [5,15,16]. However, EPC members noted that these quality assessment tools have limitations. Further, it is important to remember that these instruments do not assess the quality of primary evidence, which must also be taken into consideration. More explicit consideration of the quality rating approaches available for systematic reviews, and empiric evidence to support these, would help in the consideration and selection of existing systematic reviews for use in new reviews.

#### Determining appropriate use for relevant existing systematic reviews: scanning references

##### Available guidance

Cochrane, the EPC program, and CRD provide guidance on scanning the reference lists of existing systematic reviews (see Table 3). These organizations suggest scanning the reference lists of existing reviews as a supplementary method to find relevant studies for a new systematic review. Prior EPC program guidance suggested cross-checking primary studies from one or more existing systematic reviews to confirm adequacy of primary searches or to help select the most comprehensive existing review for incorporation.

##### Evidence-based Practice Center Discussions

Overall, most EPC members had experience using existing reviews for scanning references for relevant articles. No major concerns over the use of existing reviews in this manner were presented.

##### Assessment

Some guidance is available for using existing reviews as a potential source of relevant studies for a new review. As noted below, incorporating search results from a prior systematic review to substitute for some or all of the new review’s search efforts is different from simply scanning references as an additional source of information to support other efforts. More commonly, the latter occurs (that is, reference lists from existing reviews are used to augment or check comprehensiveness of the search yield used in the current review). In general, EPC members felt comfortable using existing reviews as a way to identify possible additional articles for a review. Thus, this does not appear to be an area where future guidance is needed except for the general methodological question of when this type of hand-searching has reached saturation (that is, when no further relevant studies are being identified and searching may stop).

#### Determining use: search strategy and results of existing searches

##### Available guidance

Cochrane and the EPC program provide guidance on using the search strategy from existing reviews (see Table 3. The EPC program recommends using part or all of the search strategy from an existing review, particularly in the case of updating. The EPC program also indicates when it is acceptable to incorporate results of searches from existing systematic reviews to locate all primary studies - that is, when the search methods (for example, search strategy, inclusion/exclusion criteria, literature screening methods) are consistent with EPC program methods for finding evidence. The EPC program recommends supplementing primary studies from the existing systematic review by performing bridge searches whenever the search date for the existing review ended more than 1 year earlier than searches for the new review are being conducted [2]. Data from primary studies gathered through these combined search efforts are then abstracted, followed by a new analysis and synthesis of data. Cochrane recommends utilizing information about search strategies from existing reviews and guidelines when possible in a new review, but does not address using search results in entirety.

##### Evidence-based practice center discussions

EPC members reported sometimes using the search strategy from an existing review, particularly when search strategies are reviewed and approved as robust by their medical librarians. EPCs also mentioned that, once they have determined it is methodologically appropriate to incorporate an existing review’s search results, they go beyond simply including the primary studies from the existing search. Instead, they check the primary studies against their inclusion/exclusion criteria, and some go further by also checking all studies from the excluded studies table in the previous systematic review, particularly in cases where there are slight differences in the questions or eligibility criteria.

##### Assessment

Some guidance is available on using the search strategy and search results from existing reviews. The EPC program provides some guidance for when to trust a search strategy from a prior review. Given the developing practice of requesting peer review for their search strategies, medical librarian review prior to incorporating an existing search strategy could be warranted [2]. Existing guidance related to when and how to incorporate the results of previous search strategies is relatively robust in the current EPC guidance; incorporation of existing search results is also the most common way EPCs report incorporating existing systematic reviews into their work. Further guidance should be based on empiric evidence to establish whether any additional value is added by 1) considering included studies from more than one existing systematic review and 2) considering excluded studies from the previous systematic review upon which the search results are being based.

#### Determining use: data abstraction

##### Available guidance

Cochrane, NICE, and the EPC program provide guidance on using the data abstraction from existing reviews (see Table 3). When using existing reviews in a new guideline, NICE recommends creating new evidence summaries but using and directly referencing the original evidence tables from the prior review. For an overview of reviews, Cochrane guidance recommends using the abstracted data presented in the existing reviews and seeking additional information from the authors of systematic reviews or from primary study authors only if necessary. Though there is ongoing work in this area such as the Systematic Review Data Repository (SRDR) project, which will likely affect how abstracted data are used in the future, current guidance from the EPC program suggests using data abstraction tables from an existing review only if the methods used are deemed to be high quality and consistent with the EPC program methods for abstracting evidence.

##### Evidence-based practice center discussions

Most EPC members had concerns over whether or not data abstracted from an existing review are trustworthy, comprehensive, or accurate. Additionally, it was often reported that people tend to have to go back and abstract at least some data themselves from the primary studies due to slightly different outcomes, missing data or improper abstraction. This is particularly true when access to full evidence tables is limited, as can be the case with some peer-reviewed articles. For any data abstraction that is incorporated, spot-checking for quality assurance was recommended, though there was no consensus on the minimum level required.

##### Assessment

Some guidance is available for using the data abstraction from existing reviews. However, there are still issues to be resolved when considering using abstracted data from an existing review. Additionally, ongoing work such as the SRDR project could obviate the need for separate guidance if pre-abstracted, high-quality data become available for many reviews. At the least, tools like SRDR will result in the need for updated guidance which incorporates such new data resources for reviewers. Guidance should specifically address various possible data abstraction scenarios. One such example is guidance related to including all versus only some of the abstracted data from an existing review. Because of different review scopes, PICOTS-SD, quality criteria of included studies, *etcetera*, systematic reviewers may determine that they will only need to use a portion of the data included in an existing review. Conversely, systematic reviewers may need to add to existing abstracted data either by abstracting additional data from previously included studies or by adding entirely new studies. Finally, as noted in the EPC discussion themes, systematic reviewers are often concerned about the trustworthiness or accuracy of abstracted data. Therefore, guidance is needed to establish consistent, reliable methods for conducting data checking or confirming the abstracted data to assure accuracy.

#### Determining use: study-level risk of bias assessments

##### Available guidance

Guidance is available on assessing the risk of bias of individual studies, though little guidance specifically addresses how to incorporate this information from existing reviews into a new systematic review. AS noted in Table 3, Cochrane recommends conducting quality assessments of evidence for all outcomes addressed in an overview of reviews and evaluating the quality assessment judgments from existing reviews to ensure consistency among included reviews. If an existing review does not include a quality assessment, Cochrane recommends performing a quality assessment of the primary studies.

##### Evidence-based practice center discussions

EPC members noted that quality assessment of a systematic review to determine appropriateness for inclusion in a new systematic review is not sufficient to represent the quality of individual studies included in the existing review. When a review is incorporated as more than a source of primary studies, it raises a number of issues about how to proceed in terms of quality rating. EPC members expressed strong concerns about relying on the risk of bias assessments from prior reviews; in particular, risk of bias assessments are known to be poorly reproducible, and often lack transparency and rationale for ratings. No guidance is available on how to integrate information from reviews that do not have risk of bias assessments, or used risk of bias assessment tools different from those used in the new review. Overwhelmingly, EPC members felt that the primary studies included in an earlier review needed to be quality rated in some way that is sufficiently consistent with that applied to newly identified primary studies included in the new review. Even if existing reviews include risk of bias assessments, an overarching theme from the discussion with EPC members was that it may be difficult for reviewers to trust the reliability of other reviewers’ risk of bias assessments. Some EPC members felt that you may be able to trust particular sources (for example, Cochrane or EPCs) using particular risk of bias assessment tools.

##### Assessment

Guidance is readily available for assessing the risk of bias of primary studies with currently available tools and checklists (for example, Cochrane, EPC program) [2,8,17,18]. However, the lack of guidance about when to accept or when to repeat such assessments when conducted by others as part of prior reviews was of particular concern to EPCs. Other issues of concern for EPCs include how to handle risk of bias assessment from prior reviews that are based on different tools from those used in a new review. Lack of clear guidance in this area is a barrier since the possible need to assess risk of bias for all primary studies introduces resource demands that reduce the likelihood of efficiency gains from incorporating existing systematic reviews.

#### Determining use: synthesis, including strength of evidence assessment

##### Available guidance

Cochrane and the EPC program provide some guidance on using the synthesis from existing systematic reviews (see Table 3). Cochrane provides guidance on using prior synthesis in an update of a review and in an overview of reviews. In an update, Cochrane recommends rerunning the quantitative synthesis if data from new studies are found. In an overview of reviews, Cochrane recommends relying on the previous analysis as much as possible and only reanalyzing data if necessary (for example, if different populations or subgroups are analyzed in different reviews). The EPC program recommends incorporating an existing review in its entirety, including evidence summaries, only if it is high quality, the key questions are very similar to the new review’s questions, and the methods used are consistent with EPC program methods. However, current EPC program guidance does not explicitly address methods for incorporating prior syntheses. The EPC program does address dealing with discordant results from existing reviews and recommends making an effort to determine reasons for the disagreements both as part of the new review planning and in discussion of its findings. At the review planning stage, uncertainty of which discrepant results to trust can provide a strong rationale for conducting a new review. No current guidance is available on performing strength of evidence assessments for systematic reviews, particularly as one component of a larger body of evidence answering a key question.

##### Evidence-based practice center discussions

Most EPC members had concerns over how and if you can trust a prior review and whether or not you can trust the data synthesis. The lack of guidance available for assessing strength of evidence when integrating an existing systematic review into a new review poses particular challenges. Despite current guidance, EPC members had additional concerns about how to deal with discordant results from reviews.

##### Assessment

Limited guidance is available on using the synthesis from existing reviews and this is an area that EPC members struggle with when considering using existing reviews. EPCs particularly struggle when asked to assess overall strength of evidence for mixed bodies of evidence (that is, those representing both primary studies and prior systematic reviews). The difficulty is compounded by the need for different guidance, depending on the circumstances surrounding the prior review(s) and the current review. We identified four scenarios that might require different guidance as to how the synthesis may be used from an existing review: 1) up-to-date and has a meta-analysis; 2) up-to-date and presents a narrative summary; 3) not up-to-date and has a meta-analysis; and 4) not up-to-date and presents a narrative summary. These scenarios obviously apply only when one or more existing reviews are being included in a new review based on meeting certain criteria (for example, scope of key questions, PICOTS, and quality).

#### Methods and results reporting

##### Available guidance

IQWiG, NICE, Cochrane and the EPC program present guidance on reporting results when utilizing existing reviews (see Table 3). The Annals of Internal Medicine has also published methodological reporting requirements for authors [19]. For a benefit assessment based on systematic reviews, IQWiG recommends summarizing the results from existing reviews in tables and noting any differences in results between reviews. In a guideline document using existing reviews, NICE recommends referencing evidence tables from existing guidelines with a direct link to the source. Cochrane provides guidance on how to present information in an update of a review, including how to highlight information from new studies. When integrating existing reviews, the EPC program recommends including a summary table of included systematic reviews, including an assessment of the overlap in primary studies between reviews. Additionally, the EPC program recommends justifying the use of existing reviews in a new review and commenting on advantages and disadvantages of using existing reviews in the discussion session. In an update of a review, the EPC program recommends explicitly showing what has changed from the previous report.

##### Evidence-based practice center discussions

EPC members had concerns about how to show evidence from reviews, including how to highlight the primary evidence when using a prior review. Challenges have surfaced when users of EPC reports are unable to identify specific details about primary studies because they have been included as a part of a systematic review instead of highlighted individually. Some EPC members have presented varying levels of detail based on how they used existing reviews but had no guidance on the best approaches. Other EPC members have presented existing systematic reviews in tables and pulled out elements for discussion, even when the existing reviews are not otherwise integrated into a report.

##### Assessment

While some guidance is available for how to report the methods to locate and decide how to use an existing review, issues still may arise when reporting results from existing reviews. Specifically, it can be difficult to highlight primary studies or isolate results from individual studies when integrating results from existing reviews. It is also important to not double count studies, such as through inclusion of primary studies also included in existing systematic reviews or by considering as separate evidence systematic reviews with overlapping primary studies. Guidance is needed on the level of detail needed on all aspects, such as how new data were incorporated into evidence tables. Future examination of various approaches recommended by different groups and development of a set of templates with worked examples could be very useful.

## Conclusions

It is considered good practice in research to explicitly consider what is already known. Specifically, there is also increased pressure to use existing systematic reviews in new reviews to leverage work already completed and thus, theoretically, reduce the amount of resources needed. Starting a new systematic review is resource-intensive, but it is unclear if the benefits of incorporating existing systematic reviews into new reviews are in some ways aspirational. This is perhaps in part because of the lack of methods for doing so.

Our workgroup was formed as part of the methods work of the AHRQ EPC program and we focused on systematic reviews of health questions. Challenges in using existing systematic reviews in new reviews, and the options to address these challenges, may differ for different kinds of systematic reviews. We would suggest that examining whether our recommendations apply in these other contexts is an area for future work. However, we feel that the concepts raised and discussed here are broadly applicable to systematic reviews in general.

There is consensus among health-focused systematic review organizations and the EPCs about some aspects of incorporating existing systematic reviews into new reviews. Best practice requires a discussion of existing systematic reviews in the introduction and discussion sections of any new review. In all reviews, it is important to discuss how the current systematic review conclusions concur or differ from previous reviews. There remain, however, challenges for review authors attempting to incorporate existing systematic reviews into new systematic reviews. The methodological steps of using existing systematic reviews, status of existing guidance, and our recommendations are summarized in Table 4.

**Table 4:** Guidance recommendations for methodological areas in using existing systematic reviews

**Methodological area**	**Guidance status**	**Recommendations/Further work needed**
Locating existing reviews	Current guidance is time intensive	Narrow search to locate only highly relevant, well-done, very recent, existing reviews; consider narrowing further to specific sources like EPC or Cochrane. Empiric study of comprehensive versus limited consideration of specific sources may be warranted. Consider how much documentation of search strategies and yields is required for transparency.
Assessing relevance	Guidance exists	Follow current guidance
Assessing review quality	Current tools, such as AMSTAR, have limitations and none consider primary literature included in the reviews	Empiric evidence of quality rating approaches is needed. Consider which currently available (or soon to be available) tools best fit the EPC program’s needs.
Determining use		
Scanning references	Guidance exists	Follow current guidance
Search strategy/results of existing searches	Guidance exists	Empiric evidence for considering searches from >1 review and considering excluded studies is needed
Data abstraction	Current guidance is limited	Guidance needed for specific scenarios and for confirming accuracy of abstracted data
Study-level risk of bias assessments	Guidance available for primary studies	Guidance is needed for when to accept or repeat assessments from existing reviews
Synthesis	Current guidance is limited	Guidance needed for specific scenarios and for assessing strength of evidence when integrating existing review
Methods and results reporting	Guidance exists	Guidance needed on level of detail necessary for all aspects, and options with worked examples needed for evidence tables

AMSTAR, A Measurement Tool to Assess Systematic Reviews; EPC, Evidence-based Practice Center.

Remaining areas of uncertainty may be addressed through a variety of methods. For instance, how best to construct evidence tables that combine primary studies and evidence from existing reviews may be addressed through discussions with end users of systematic reviews. Methods research could assess the minimum quality control needed for using prior data abstractions or the impact on conclusions of a search of systematic reviews limited to specific sources. Finally, a symposium of systematic review organizations may be needed to develop consensus on reporting standards for communicating how prior reviews are incorporated. Future EPC program workgroups are taking up this challenge.

## Appendix A. Participating Evidence-based Practice Center members

Brown University Center for Evidence-based Medicine

Issa Dahabreh, MD, MS

Associate Director

ECRI Institute

Karen Schoelles, MD, SM, FACP

Director

Jonathan Treadwell, PhD

Associate Director

Wendy Bruening, PhD

Senior Research Analyst

Johns Hopkins University

Renee Wilson, MS

Project Manager

Lisa Wilson, ScM

Project Manager

Kaiser Permanente Research Affiliates

Jennifer Lin, MD, MCR

Associate Director

Minnesota Evidence-based Practice Center

Mary Butler, PhD, MBA

Associate Director

Michelle Brasure, PhD

Project Director

Pacific Northwest Evidence-based Practice Center

Roger Chou, MD, FACP

Director

Southern California Evidence-based Practice Center - RAND

Margaret Maglione, MPP, BA

Associate Director

RTI-UNC

Nancy Berkman, PhD

Methodologist

University of Alberta

Lisa Hartling, BScPT, MSc, PhD

Director

Donna Dryden, PhD

Associate Director

Vanderbilt University Medical Center

Nila Sathe, MA, MLIS

Program Manager

## Abbreviations

AHRQ: Agency for Healthcare Research and Quality; AMSTAR: A Measurement Tool to Assess Systematic Reviews; CDSR: Cochrane Database of Systematic Reviews; CRD: York Centre for Reviews and Dissemination; DACEHTA: Danish Centre for Health Technology Assessment; DARE: Database of Abstracts of Reviews of Effects; ECHTA: European Collaboration for Health Technology Assessment; EPC: Evidence-based Practice Center; IOM: Institute of Medicine; IQWiG: Institute for Quality and Efficiency in Health Care; NICE: National Institute for Health and Care Excellence; PICOTS-SD: Population: Intervention: Comparator: Outcome: Time Frame: Setting: and Study Design; SRDR: Systematic Review Data Repository.

## Competing interests

The authors declare that they have no competing interests.

## Authors’ contributions

KR participated in conception and design of the project, conducting interviews and drafting of the manuscript. EW participated in conception and design of the project and drafting of the manuscript. MO analyzed and interpreted project material and participated in drafting of the manuscript. JA helped analyze project material and participated in drafting of the manuscript. LH helped analyze and interpret project material and critically revised manuscript. DD helped analyze and interpret project material and critically revised manuscript. MB helped analyze and interpret project material and critically revised manuscript. SN conducted interviews, helped analyze and interpret project material and critically revised manuscript. MM conducted interviews, helped analyze and interpret project material and critically revised manuscript. NB conducted interviews, helped analyze and interpret project material and critically revised manuscript. JL critically revised manuscript. SC helped analyze and interpret project material and critically revised manuscript. All authors read and approved the final manuscript.
